# The mediating effect of body mass index on the relationship between smoking and hip or knee replacement due to primary osteoarthritis. A population-based cohort study (the HUNT Study)

**DOI:** 10.1371/journal.pone.0190288

**Published:** 2017-12-28

**Authors:** Marianne Bakke Johnsen, Alf Inge Hellevik, Milada Cvancarova Småstuen, Arnulf Langhammer, Ove Furnes, Gunnar Birkeland Flugsrud, Lars Nordsletten, John Anker Zwart, Kjersti Storheim

**Affiliations:** 1 Research and Communication Unit for Musculoskeletal Health, Oslo University Hospital, Oslo, Norway; 2 Faculty of Medicine, University of Oslo, Oslo, Norway; 3 Division of Orthopaedic Surgery, Oslo University Hospital, Oslo, Norway; 4 The HUNT Research Centre, Department of Public Health and General Practice, NTNU, Norwegian University of Science and Technology, Levanger, Norway; 5 Oslo and Akershus University College, Oslo, Norway; 6 The Norwegian Arthroplasty Register, Department of Orthopaedic Surgery, Haukeland University Hospital, Bergen, Norway; 7 Department of Clinical Medicine, University of Bergen, Bergen, Norway; Monash University, AUSTRALIA

## Abstract

To investigate the total effect of smoking on total hip or knee replacement (THR/TKR) due to primary osteoarthritis (OA) and to quantify the indirect effect of smoking through body mass index (BMI). Participants from the Nord-Trøndelag Health Study (the HUNT Study) were linked to the Norwegian Arthroplasty Register to detect the first THR or TKR due to primary OA. A mediation analysis was used to decompose the total effect of smoking into a direct and indirect effect. BMI was considered a mediator in the analysis. All effects were estimated as hazard ratios (HRs) with 95% confidence intervals (CIs). The indirect effect of smoking mediated through BMI was expressed as a percentage (proportion*100). In total 55 188 participants were followed up during 17.2 years (median). We identified 1322 THRs and 754 TKRs. For men, the total effect of current vs. never smoking revealed a decreased risk of THR (HR 0.59, 95% CI 0.46–0.76) and TKR (HR 0.47, 95% CI 0.32–0.66). For women, current smoking increased the risk of THR (HR 1.34, 95% CI 1.11–1.60). For men, 6% and 7% of the risk reduction for THR and TKR, respectively, was mediated by BMI. We found a negative association between smoking and THR or TKR for men. On the contrary, smoking was associated with increased risk of THR for women. Most of the effect of smoking on joint replacement risk remained unexplained by BMI.

## Introduction

Hip and knee osteoarthritis (OA) has been ranked the 11^th^ highest contributor to global disability [1]. Known risk factors include older age, being overweight or obese, female gender, previous joint injury and heavy physical workload [2–6]. The association between smoking and OA has been inconclusive. It has previously been suggested that smoking has a protective effect on knee OA [7, 8] and recent studies have confirmed this protective effect for total hip (THR) and knee replacement (TKR) [5, 9, 10]. Results from in vitro data have indicated that any protective effect may be related to the beneficial effect of nicotine on chondrocyte function [11, 12], however, studies which have assessed the effect on articular cartilage volume using MRI have shown conflicting results [13–15]. Moreover, a possible explanation for the inverse association is smoking’s connection with other lifestyle factors that may affect the risk-relationship with OA. Increased weight or body mass index (BMI) is an established risk factor for OA and subsequent joint replacement [6, 16, 17]. If smoking is associated with low BMI, as corroborated in Mendelian randomization studies [18, 19], then part of the protective effect of smoking may be due to BMI. Further, if BMI is one of the biological mechanisms through which smoking can affect the risk of OA (i.e. indirect effect), then BMI should be considered as a mediator in the total effect of smoking on OA [20]. To assess mediation, an approach where the total effect is decomposed into a direct and an indirect effect can be used [21]. Thus, the main objectives of the present study were; 1) to investigate the total effect of smoking on the risk of THR or TKR due to primary OA, and 2) to quantify the proportion of the total effect of smoking that was mediated through BMI.

## Materials and methods

### Study population

The Nord-Trøndelag Health Study (HUNT) is a large population-based database collected through three surveys; HUNT1 (1984–1986), HUNT2 (1995–1997) and HUNT3 (2006–2008). All residents of Nord-Trøndelag County in Norway aged 20 years and older were invited to participate. A wide range of health related topics were addressed, as previously described [22]. In HUNT2, 93 898 residents were invited to take part. Of these, 64 978 (69.2%) participated in both answering questionnaires and attending clinical examination, the data from which we included in the present study. We excluded participants due to joint replacement prior to baseline (n = 833), no date recorded for the first joint replacement (n = 172), age ≥ 80 years at baseline (n = 2579), self-reported OA at baseline (n = 5141), missing data on smoking status (n = 1063) or due to emigration before start of follow-up (n = 2). Our study sample therefore comprised 55 188 participants.

### Exposure

Smoking status was categorised into never, former and current smokers based on answers to the questionnaire at baseline in HUNT2.

### Outcome

The outcome of interest was the first THR or TKR due to primary OA. The unique 11-digit identity numbers of Norwegian citizens enabled us to link individuals’ data in HUNT2 with data in the Norwegian Arthroplasty Register (NAR) to prospectively detect THRs or TKRs. Joint replacements due to conditions other than primary OA were censored. Patients with more than one THR or TKR were only counted once. The time frame of data included from NAR was from September 15, 1987 (origin of NAR) until December 31, 2013. The completeness of THR and TKR registration in NAR is >95% [23, 24].

### Covariates

Age at baseline in HUNT2, gender, education (<10, 10–12 and ≥13 years), physical activity (PA), and work status (employed/unemployed) were considered as confounders based on previous studies and a priori reasoning. Education and work status were included as proxies for socioeconomic status. PA during the last year was self-reported as light (no sweating or shortness of breath) and/or vigorous (sweating or short of breath) with four options of duration (0, <1, 1–2, ≥3 hours per week). PA was further classified into inactive (no light or vigorous PA), low (<3 hours of light, and no vigorous PA), moderate (≥3 hours of light and/or <1 hour of vigorous PA) and high PA (≥1 hour of vigorous, regardless of any light PA), as previously described for the cohort [25]. Diabetes and cardiovascular disease (CVD) and were considered as potential confounders. Diabetes, myocardial infarction (MI), angina pectoris and stroke/brain hemorrhage were defined by affirmative answers to the questions “Have you had, or do you have any of the following: diabetes, MI, angina and/or stroke?” CVD was defined as a composite of MI, angina or stroke [26]. Height and weight were measured by trained personnel at baseline in HUNT2. BMI is weight in kilograms divided by height in meters squared.

### Statistical analyses

**Fig 1:**
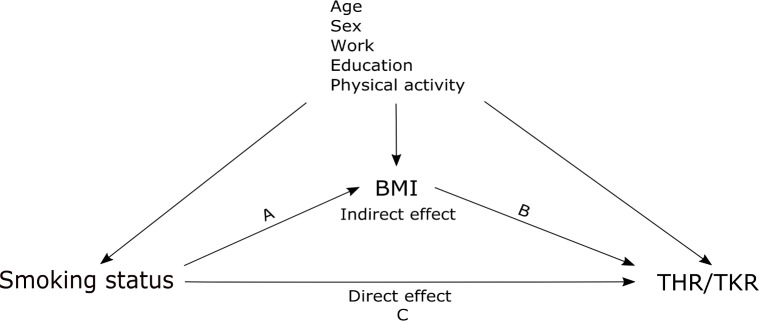
Model of the potential mediating effect of body mass index (BMI) on the relationship between smoking status and hip or knee replacement (THR/TKR). The total effect of smoking includes the product of the direct (C) and indirect (AB) effects.

Descriptive statistics are given as means and standard deviation (SD) or percentages. Start of follow-up was the date of inclusion in HUNT2 (in 1995–97). The participants were followed until date of THR/TKR due to primary OA, date of THR/TKR for conditions other than primary OA, date of death/emigration, or the end of follow-up (December 31, 2013), whichever came first. Diabetes and CVD were tested as possible confounders; however they did not affect the magnitude or the direction of association between smoking and THR or TKR. Hence, they were not included in further analyses to increase statistical power. The final model included adjustments for age (as the time scale), sex, PA, work status and education. BMI was considered a mediator and included as a continuous variable. The underlying model of our analyses is illustrated in Fig 1.

Mediation analysis was performed in order to decompose the total effect of smoking into a direct and an indirect effect [21]. Regression parameters for the direct and indirect effects were obtained in two stages. Firstly, through a linear regression of BMI on smoking (Fig 1, path A) and secondly, through a Cox proportional hazards regression model of THR/TKR on smoking and BMI (Fig 1, path B and C). The total effect of smoking comprised the product of the direct (C) and indirect (AB) effects. We adjusted for the selected confounders in both models. Ultimately all effects were estimated as hazard ratios (HR) with 95% confidence intervals (CI). The proportional hazards assumptions were tested in each of the models using Schoenfeld residuals. P-values <0.05 were considered statistically significant.

The proportion of the total effect of smoking mediated through BMI was calculated on the ln(HR) scale and given as a percentage: ln(HR_indirect effect_) / ln(HR_total effect_)*100. We used bootstrapping with 5000 iterations to calculate the 95% CIs. To assess whether the association between smoking and joint replacement was modified by sex or age, interaction terms between smoking status and age or sex were included in the model and tested with the likelihood ratio test. There was evidence of effect modification by sex for THR (p<0.001) and TKR (p = 0.04), thus all models were stratified by sex.

We performed a sensitivity analysis on the full data set, including only age (as the time scale), sex (stratified on) and BMI (as the mediator), due to high numbers of missing values in one of the covariates (PA). Furthermore, we performed a sensitivity analysis including those with prevalent OA at baseline in the fully adjusted model to assess the potential risk of selection bias. Smokers have increased risk of mortality compared to non-smokers thus cumulative incidences for THR and TKR were calculated and depicted using the Fine and Grey approach, including mortality as the competing event [27]. All statistical analyses were performed using Stata 13.0/IC (StataCorp LP, College Station, TX, USA) and R, version 3.2.0.

### Ethics

The HUNT Study and NAR have been approved by the Regional Committee for Medical Research Ethics (REK) and the Data Inspectorate of Norway, and all participants have signed a written informed consent which also includes consent to linkage with other registries. For the current study, approval was obtained from REK Sør-Øst C 2013/151.

## Results

A total of 26 483 men and 28 394 women were included in the analyses. We identified 828 (2.9%) and 490 (1.9%) THRs in women and men, respectively, during follow-up of 17.2 years (median). Correspondingly, we identified 457 (1.6%) TKRs in women and 296 (1.1%) TKRs in men. The highest incidence of THRs and TKRs were among former smokers (Table 1). Mean age (SD) at THR was 68.9 (9.3) years and 68.2 (9.3) years at TKR. Women accounted for 62% of the joint replacements. A greater proportion of women (never, former and current smokers) were of normal weight, while in comparison a larger proportion of men were overweight. For all categories of smoking status, a higher proportion of men, compared with women, engaged in high PA, although a larger proportion of these men had prevalent CVD and diabetes at baseline (Table 1).

**Table 1:** Baseline characteristics of the study participants according to smoking status and gender.

		Smoking status women			Smoking status men	
	Never (n = 13 151)	Former (n = 6324)	Current (n = 8919)	Never (n = 10 446)	Former (n = 8517)	Current (n = 7520)
Age in HUNT*, **years, mean (SD)**	47.1 (17.3)	47.8 (13.8)	44.1 (13.3)	41.9 (14.5)	54.3 (14.4)	47.2 (14.3)
Age at THR**/TKR***, **years, mean (SD)**	70.2 (9.3)	68.5 (9.2)	65.2 (8.9)	66.7 (9.0)	70.5 (9.0)	67.4 (9.8)
**Number of THR/TKR**	399/233	217/131	212/93	184/109	209/141	97/46
BMI****, **kg/m**^**2**^**, mean (SD)**	26.2 (4.5)	26.5 (4.5)	25.2 (4.2)	26.2 (3.4)	27.2 (3.4)	25.9 (3.6)
**Underweight (<18.50), %**	0.7	0.6	1.8	0.2	0.1	0.7
**Normal (18.50–24.99), %**	43.8	40.9	53.1	38.2	25.8	41.6
**Overweight (25.00–29.99), %**	36.8	39.9	32.3	49.0	55.7	45.4
**Obese (≥30.00), %**	18.0	18.0	12.5	12.3	17.9	12.1
**Missing**	0.7	0.7	0.3	0.3	0.5	0.2
**Education, %**						
**<10 years**	54.6	65.5	73.4	57.7	72.0	76.7
**10–12 years**	12.8	10.7	10.6	11.7	5.7	6.59
**≥13 years**	27.7	20.2	12.8	28.1	17.8	12.8
**Missing**	5.0	3.6	3.2	2.4	4.6	4.0
**Work status, %**						
**Unemployed**	20.0	17.2	15.8	12.6	32.2	23.7
**Employed**	77.7	81.0	82.1	86.2	66.2	74.6
**Missing**	2.3	1.8	2.1	1.2	1.5	1.7
**Physical activity, %**						
**Inactive**	4.9	5.7	7.8	6.4	7.4	10.3
**Low**	16.9	18.3	20.2	12.2	14.5	15.9
**Moderate**	31.2	32.8	30.9	29.5	31.9	30.9
**High**	23.6	21.5	18.0	40.6	27.5	23.4
**Missing**	23.0	21.8	23.2	11.4	18.7	19.6
CVD*****, **%**	4.2	4.6	2.8	3.2	15.1	6.8
**Missing**	0.3	0.2	0.2	0.2	0.2	0.2
**Diabetes, %**	2.6	2.3	1.2	1.9	4.1	2.0
**Missing**	0.2	0.1	0.1	0.1	0.3	0.2

*HUNT = The Nord-Trøndelag Health Study

**THR = total hip replacement

***TKR = total knee replacement

****BMI = body mass index (BMI categories are defined by the WHO cut-off points)

*****CVD = cardiovascular disease (composite of myocardial infarction, angina pectoris or stroke).

All differences among never, former and current smokers were statistically significant (p<0.001)

### Total and indirect effects of smoking on THR

For men, the total effect of both current and former vs. never smoking revealed a decreased risk of THR with HR 0.59 (95% CI 0.46–0.76) and HR 0.69 (95% CI 0.57–0.85), respectively (Table 2). The indirect effect of current smoking through BMI also reduced the risk (HR_IE_ 0.97, 95% CI 0.96–0.98), while the indirect effect of former smoking contributed in the opposite direction (HR_IE_ 1.07, 95% CI 1.05–1.10). For women, the total effect of current vs. never smoking was associated with an increased risk of THR (HR 1.34, 95% CI 1.11–1.60), while the indirect effect reduced risk (HR_IE_ 0.94, 95% CI 0.93–0.96). The total effect of former smoking was not statistically significant for women (Table 2).

**Table 2:** Total, direct, and indirect effects of smoking on the risk of hip replacement (THR) by smoking status, adjusted for age, work status, physical activity and education.

	Men THR		Women THR	
	HR (95% CI)^a^	Proportion^b^ mediated (%) (95% CI)^a^	HR (95% CI)^a^	Proportion^b^ mediated (%) (95% CI)^a^
**Effects current vs.** **never smokers**	THR current = 76^c^ vs. THR never = 147^c^		THR current = 146^c^ vs. THR never = 246^c^	
**Total effect**	0.59 (0.46–0.76)	100%	1.34 (1.11–1.60)	100%
**Direct effect**	0.61 (0.47–0.79)	94% (88%-97%)	1.42 (1.17–1.71)	120% (111%-154%)
**Indirect effect via BMI**	0.97 (0.96–0.98)	6% (3%-13%)	0.94 (0.93–0.96)	-20% (-54%; -11%)
**Effects former vs.** **never smokers**	THR former = 165 ^c^ vs. THR never = 147 ^c^		THR former = 139 ^c^ vs. THR never = 246 ^c^	
**Total effect**	0.69 (0.57–0.85)	100%	1.17 (0.97–1.14)	100%
**Direct effect**	0.65 (0.53–0.80)	119% (110%-144%)	1.15 (0.96–1.38)	89% (20%-162%)
**Indirect effect via BMI**	1.07 (1.05–1.10)	-19% (-44%; -11%)	1.02 (1.01–1.03)	11% (-62%-80%)

HR = hazard ratio, CI = confidence interval, BMI = body mass index.

^a^: Bootstrapping with 5000 iterations was used to calculate the uncertainty of the estimates.

^b^: On ln(HR) scale.

^c:^ The number of current, former or never smokers with THR.

### Proportions mediated by BMI for THR

For men, 6% (95% CI 3%-13%) of the effect of current smoking was mediated by BMI, i.e. 6% of the risk difference between current and never smoking was explained by BMI (Table 2). For women, a negative proportion was mediated in the comparison of current and never smokers (-20%, 95% CI -54%; -11%) due to the opposite directions of the direct and indirect effects. Similar results were found for former vs. never smoking in men (-19%, 95% CI -44%; -11%). As the total effect of former smoking was not statistically significant for women, the associated proportion mediated was not considered (Table 2).

### Total and indirect effects of smoking on TKR

For men, the total effect of current vs. never smoking revealed a decreased risk of TKR (HR 0.47, 95% CI 0.32–0.66), while the effect of former smoking was not significant (Table 3). The indirect effect of current smoking through BMI also reduced the risk (HR_IE_ 0.95, 95% CI 0.93–0.96). For women, the total effects of current and former vs. never smoking on TKR were not statistically significant (Table 3).

**Table 3:** Total, direct, and indirect effects of smoking on the risk of knee replacement (TKR) by smoking status, adjusted for age, work status, physical activity and education.

	Men TKR		Women TKR	
	HR (95% CI)^a^	Proportion^b^ mediated (%) (95% CI)^a^	HR (95% CI)^a^	Proportion^b^ mediated (%) (95% CI)^a^
**Effects current vs.** **never smokers**	TKR current = 35^c^ vs. TKR never = 87 ^c^		TKR current = 64 ^c^ vs. TKR never = 144^c^	
**Total effect**	0.47 (0.32–0.66)	100%	0.95 (0.72–1.21)	100%
**Direct effect**	0.49 (0.33–0.69)	93% (87%-96%)	1.09 (0.83–1.39)	-^d^
**Indirect effect via BMI**	0.95 (0.93–0.96)	7% (4%-13%)	0.87 (0.85–0.89)	-^d^
**Effects former vs.** **never smokers**	TKR former = 108 ^c^ vs. TKR never = 87 ^c^		TKR former = 80 ^c^ vs. TKR never = 144 ^c^	
**Total effect**	0.82 (0.64–1.06)	100%	1.26 (1.00–1.57)	100%
**Direct effect**	0.73 (0.57–0.94)	-^d^	1.21 (0.97–1.50)	83% (10%-95%)
**Indirect effect via BMI**	1.12 (1.10–1.15)	-^d^	1.04 (1.02–1.06)	17% (5%-90%)

HR = hazard ratio, CI = confidence interval, BMI = body mass index.

^a^: Bootstrapping with 5000 iterations was used to calculate the uncertainty of the estimates.

^b^: On ln(HR) scale.

^c:^ The number of current, former or never smokers with TKR.

^d^: Percentages as proportion of the total effect are not given. Estimates were numerically unstable and therefore meaningless due to division by numbers (ln(HR_total effect_)) close to zero.

### Proportions mediated by BMI for TKR

For men, 7% (95% CI 3%-13%) of the risk difference between current and never smoking was explained by BMI. For women, the total effect of former smoking was not statistically significant therefore we did not consider the associated proportion mediated. Moreover, the proportions mediated related to former smoking in men and current smoking in women could not be calculated due to numbers (ln(HR_total effect_)) close to zero (Table 3).

The sensitivity analysis on the full data set revealed no change in the total effect of smoking in men. For women, the magnitude of total effects was somewhat larger and former smoking was significantly associated with THR and TKR (S1 and S2 Tables). Furthermore, including those with prevalent OA in the model did not change either the size or level of statistical significance of the total effects of smoking (data not shown). The cumulative incidences of THR and TKR, accounting for mortality, were small in terms of absolute numbers. As anticipated, our data confirmed a higher cumulative mortality for current smokers compared to never and former smokers, especially in men (S1 Fig B and S2B Fig).

## Discussion

Our analysis of a large population-based cohort supported previous findings of a negative association between smoking and THR and TKR in men. In contrast, we found smoking to increase the risk of THR in women. The indirect effects of smoking mediated by BMI were small. To our knowledge, this is the first study to investigate the indirect effects of BMI in the context of smoking and subsequent THR or TKR.

The main strengths of our study are the prospective design, data from a large well-characterized cohort and the length of follow-up. Our case ascertainment of THR and TKR through linkage with the nationwide register ensured nearly complete data on joint replacements [23, 24]. Our study sample comprised a wide age range, including a large number of participants in the age at risk for OA and joint replacement. We excluded those with prevalent OA at baseline in order to assess both incident cases of OA and THR/TKR in a population at risk. This might have introduced selection bias, since OA may serve as an intermediate step between smoking and THR/TKR, however, the sensitivity analysis did not suggest this.

A limitation is that smoking status was self-reported and we had no data of change in smoking habits during follow-up, which may have resulted in misclassification of exposure. Still, misclassification of smoking should be non-differential due to the prospective design of this study; if it did have any effect it would be to weaken the association. Smoking and BMI were both measured at baseline in HUNT. Therefore, the time sequence between the exposure and the mediator may not be clear and the observed associations might not reflect actual causal associations.

Joint replacement was used as a proxy for severe hip and knee OA. The limitation of this case definition is that it only covers those being surgically treated. Relying on the number of THRs and TKRs alone may lead us to underestimate the true burden of the disease. Moreover, the general health status of the patient has an influence on the orthopaedic surgeon’s choice regarding treatment, possibly giving a “healthy patient” selection bias with corresponding underestimation of the association between smoking and severe OA. In line with this, we excluded those ≥80 years old at baseline and included all-cause mortality as the competing event when presenting the cumulative incidences. Assumptions of no confounding have to be made for the mediation model [21, 28]. Still, we cannot be sure that there is no bias. Therefore, inferences about causality cannot be made based on the given study design alone.

The total effect of current smoking in men revealed that the risk of THR was reduced by 41% and the risk of TKR by 53% compared to never smoking. For men, the reduced risk was also present among former smokers, although former smokers may represent a more heterogeneous group with regard to reasons for and time since smoking cessation. Contrary to men, current smoking in women was associated with a 34% increase in the risk of THR. Further, there was an indication of increased risk among former smokers for TKR (HR 1.26, 95% CI 1.00–1.57, p = 0.0496). However, this finding was considered to be non-significant due to the size of our dataset and the number of tests we performed. The indication of an increased risk of TKR among former smokers (women) might be more attributed to the indirect effect through BMI, as the direct effect of former smoking was non-significant. This corresponds to the fact that former smokers tend to gain weight compared to current smokers [19], and increased body weight is an established risk factor for TKR [16, 17]. This corroborates with the findings from a population-based cohort of 63 257 Chinese men and women, where the inverse association between smoking and risk of TKR was quickly attenuated with increasing duration of smoking cessation [9]. Our findings for men are in line with results from Leung et al. [9]. They found that men had a 60% lower risk of TKR associated with current vs. never smoking. However, contrary to our results, they found current smoking to be protective of TKR in women as well [9]. They adjusted for BMI, however BMI was self-reported which has been shown to be prone to underreporting, especially in those with OA [29].

Similarly, an Australian cohort study reported that men and women who were smokers were respectively 40% and 30% less likely to undergo a THR or TKR [10]. However, the participants were much older (mean age 73 at baseline) than in our study (mean age 47.0 at baseline). Consequently, it is difficult to compare results between the Australian study and our own due to the differences in methods and study samples. In addition, the qualitative measure of current smoking might reflect different intensity and duration of exposure across study populations and comparison of any results should be viewed in light of this limitation.

Similar to us, a Swedish case-control study of women (ages 50–70 years) found an increased relative risk of THR for smokers and ex-smokers compared to never smokers after adjusting for age, BMI, parity, sports activities, workload, and use of contraceptive pills and estrogen substitution [30]. The interaction between smoking and sex in our data may be modified by sex hormones, e.g. estrogen, especially after menopause in women. This could have an effect on bone mass and articular cartilage and thereby explain some of the increased risk of OA in women [31]. However, this theory is still speculative.

Correspondingly, there is no clear biological explanation for the inverse association between smoking and OA, but one theory is that any protective effect of smoking on OA may be related to the upregulation of glycomsaminoglycan and collagen synthetic activity of articular chondrocytes as a direct effect of nicotine, which has been shown in vitro [11]. These findings have been replicated in a study on articular chondrocytes from OA patients [12]. In contrast, components of tobacco smoke have shown to have a detrimental effect on chondrocyte function in intervertebral discs [32]. However, these conflicting results are from experimental studies and need to be further investigated in vivo.

Another possible explanation for the protective effect of smoking is its connection to other lifestyle factors, e.g. BMI, that affect the smoking-OA relationship. The total effect of smoking on OA is the effect of all potential causal pathways. Thus, as proposed by Felson and Zhang [20], not adjusting for BMI may actually provide a better estimate if we are interested in the total effect of smoking. Other studies have generally adjusted for BMI [5, 9, 10], and therefore not directly comparable to our results based on models including BMI as a mediator.

For men, 6% and 7% of the reduced risk (total effect of smoking) for THR and TKR, respectively, was mediated through BMI. The mediation that we observed indicated that most of the smoking effect remained unexplained by BMI. This means that the effect could either be attributed to smoking itself (direct effect), to other causal pathways not known or addressed in our analysis or to residual confounding [28]. We have to emphasize that this study focused on the effect of smoking through BMI, and is not to be interpreted as the risk of BMI alone, already a well-established risk factor for OA.

A drawback of the proportion mediated is that it is an unstable measure. This can lead to a proportion mediated larger than 100%, which is not really meaningful. Thus, it is advisable to use the proportions mediated only when the direct and indirect effects operate in the same direction [33]. The negative proportions we found regarding former smoking and THR for men and current smoking and THR for women were a result of direct and indirect effects which operated in opposite directions. Therefore, we did not include any further interpretation of these proprotions. Similarly, the proportions mediated for former smoking in women were not considered as the associated total effects of smoking was not statistically significant.

It is challenging to give a comprehensive explanation to the mechanisms of the opposite effects of smoking among men and women, especially since previous studies have predominantly found similar effects for both genders [8–10]. Higher smoking quantity has been associated with higher comorbidity and mortality, and tobacco smoking has shown to contribute more to the burden of disease in men than in women [34, 35]. Comorbidities like CVD still have low levels of evidence for being risk factors for OA [2]. Including CVD and diabetes in our statistical model did not affect the association between smoking and THR/TKR. We could have considered CVD and diabetes as mediators in the total effect of smoking on THR and TKR risk. However, this line of investigation would have been based upon an assumption of a causal relationship between CVD/diabetes and THR or TKR that may not yet be well established.

Mortality was almost twice as high for male smokers compared to female smokers in our study. The cumulative incidences of THR or TKR, accounting for mortality as the competing event, showed that current smokers were less likely to receive a joint replacement compared with former and never smokers. However, the relative risk (normally derived HR) is still a valid measure of the association between smoking and THR/TKR at a given time for those who have not yet experienced the outcome of interest or the competing event. Therefore, we did not calculate the subhazard ratio according to Fine and Grey [27] to use in the mediation analysis, as the main focus of the study was to measure the association between smoking and THR/TKR in those at risk of the main event, regardless of any competing events. In situations where the etiology of disease is of interest, the cause-specific (normally derived) HR is suggested to be an appropriate and valid measure of relative risk [36].

Moreover, women may be more likely to self-report OA and OA pain [37, 38]. Thus, the positive association between smoking and THR for women in our study may have also been influenced by non-biological factors such as health seeking behavior, reporting of symptoms and willingness to undergo surgery.

Despite opposite total effects for men and women, the indirect effects of smoking through BMI were the same. For example, the indirect effect of current smoking via BMI contributed to a reduced risk of THR and TKR in both genders (HR_IE_ <1.00), regardless of whether the total effect was protective or not. Thus, the gender differences were not related to different indirect effects in our sample, but rather to other unknown or uninvestigated confounders or mediators. We investigated only a single mediator and included well-known confounders to the smoking-severe OA relationship. However, mechanistic explanation in biology consists of complex pathways where the effects are most certainly mediated along various paths.

The participation rate in the HUNT2 survey was fairly high (>69%) [39]. The lowest participation rate was found amongst men in the youngest age group (20–29 years). Participants who were non-responders to smoking status in our study were most often women, obese, unemployed or missing data on work status and/or PA and had higher incidents of comorbidity compared to those included in the analyses. Therefore, our study sample may not represent the youngest men and those with a more complex health status.

## Conclusions

Our findings demonstrated a negative association between current smoking and the risk of THR and TKR among men. In contrast, current smoking was associated with increased risk of THR for women. Most of the smoking effect remained unexplained by BMI. The opposite total effects of smoking between genders may be explained to some extent by differences in comorbidity and mortality. Further studies are needed to disentangle the effect of smoking on severe OA.

## Supporting information

S1 Fig A and B cumulative incidences of hip replacement (THR) accounting for the competing event of death in women and men, respectively.
(TIF)

S2 Fig A and B cumulative incidences of knee replacement (TKR) accounting for the competing event of death in women and men, respectively.
(TIF)

S1 Table Total, direct, and indirect effects of smoking on the risk of hip replacement (THR) by smoking status, adjusted for age. (DOCX)

S2 Table Total, direct, and indirect effects of smoking on the risk of knee replacement (TKR) by smoking status, adjusted for age. (DOCX)
