# Supplementary figures and images for: The mediating effect of body mass index on the relationship between smoking and hip or knee replacement due to primary osteoarthritis. A population-based cohort study (the HUNT Study)
Source: PLoS One. 2017 Dec 28;12(12):e0190288. doi: 10.1371/journal.pone.0190288 (PMC5746263; doi:10.1371/journal.pone.0190288)

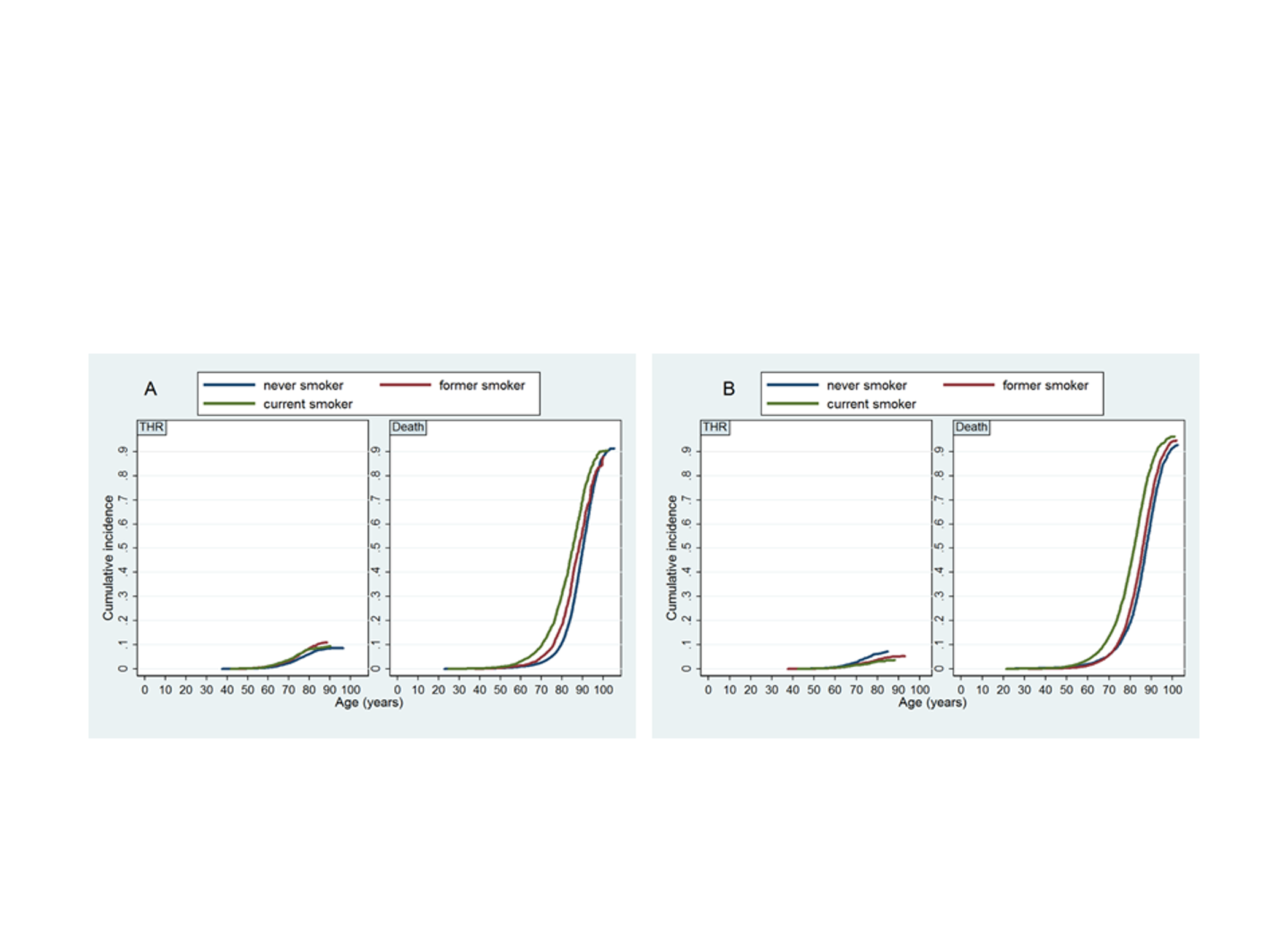

Supplement: S1 Fig — A and B cumulative incidences of hip replacement (THR) accounting for the competing event of death in women and men, respectively. (TIF) [file pone.0190288.s001.tif]

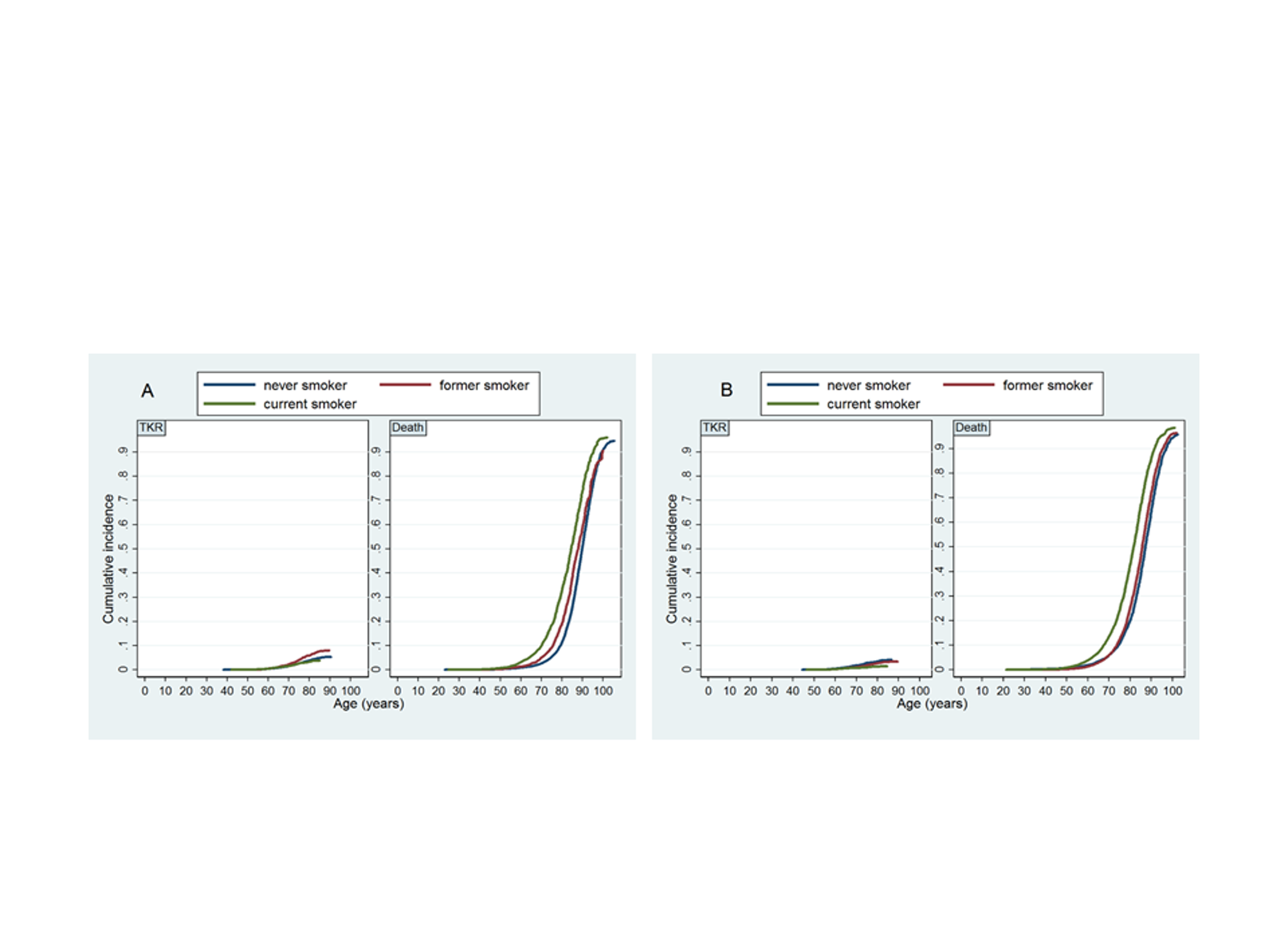

Supplement: S2 Fig — A and B cumulative incidences of knee replacement (TKR) accounting for the competing event of death in women and men, respectively. (TIF) [file pone.0190288.s002.tif]
