# Supplementary material for: The mediating effect of body mass index on the relationship between smoking and hip or knee replacement due to primary osteoarthritis. A population-based cohort study (the HUNT Study)
Source: PLoS One. 2017 Dec 28;12(12):e0190288. doi: 10.1371/journal.pone.0190288 (PMC5746263; doi:10.1371/journal.pone.0190288)
Supplement: S1 Table — (DOCX) [file pone.0190288.s003.docx]

S1Table. Total, direct, and indirect effects of smoking on the risk of hip replacement (THR) by smoking status, adjusted for age.

|  | Men THR | | Women THR | |
| --- | --- | --- | --- | --- |
|  | HR (95% CI)^a^ | Proportion^b^ mediated (%) (95% CI)^a^ | HR (95% CI)^a^ | Proportion^b^ mediated (%) (95% CI)^a^ |
| Effects current vs.  never smokers | THR current=99^c^ vs. THR never=184^c^ | | THR current=214^c^ vs. THR never=399^c^ | |
| Total effect | 0.60 (0.47-0.77) | 100% | 1.47 (1.20-1.78) | 100% |
| Direct effect | 0.61 (0.47-0.79) | 96% (90%-98%) | 1.53 (1.23-1.85) | 110% (105%-118%) |
| Indirect effect via BMI | 0.98 (0.97-0.99) | 4% (2%-10%) | 0.96 (0.95-0.98) | -10% (-18%; -5%) |
|  |  |  |  |  |
| Effects former vs.  never smokers | THR former=209^c^ vs. THR never=184^c^ | | THR former=217^c^ vs. THR never=399^c^ | |
| Total effect | 0.70 (0.60-0.89) | 100% | 1.31 (1.09-1.58) | 100% |
| Direct effect | 0.65 (0.56-0.83) | 121% (113%-171%) | 1.29 (1.07-1.55) | 93% (80%-97%) |
| Indirect effect via BMI | 1.08 (1.06-1.10) | -21% (-71%; -13%) | 1.02 (1.01-1.03) | 7% (3%-20%) |

HR=hazard ratio, CI=confidence interval, BMI=body mass index.

^a^: Bootstrapping with 5000 iterations was used to calculate the uncertainty of the estimates.

^b^: On ln(HR) scale.

^c:^ The number of current, former or never smokers with THR.
