# Supplementary material for: The mediating effect of body mass index on the relationship between smoking and hip or knee replacement due to primary osteoarthritis. A population-based cohort study (the HUNT Study)
Source: PLoS One. 2017 Dec 28;12(12):e0190288. doi: 10.1371/journal.pone.0190288 (PMC5746263; doi:10.1371/journal.pone.0190288)
Supplement: S2 Table — (DOCX) [file pone.0190288.s004.docx]

S2 Table. Total, direct, and indirect effects of smoking on the risk of knee replacement (TKR) by smoking status, adjusted for age.

|  | Men TKR | | Women TKR | |
| --- | --- | --- | --- | --- |
|  | HR (95% CI)^a^ | Proportion^b^ mediated (%) (95% CI)^a^ | HR (95% CI)^a^ | Proportion^b^ mediated (%) (95% CI)^a^ |
| Effects current vs.  never smokers | TKR current=47^c^ vs. TKR never=109^c^ | | TKR current=93^c^ vs. TKR never=233^c^ | |
| Total effect | 0.49 (0.32-0.63) | 100% | 1.06 (0.84-1.39) | 100% |
| Direct effect | 0.50 (0.33-0.66) | 95% (91%-98%) | 1.17 (0.93-1.53) | -^d^ |
| Indirect effect via BMI | 0.97 (0.94-0.98) | 5% (2%-9%) | 0.91 (0.89-0.93) | -^d^ |
|  |  |  |  |  |
| Effects former vs.  never smokers | TKR former=141^c^ vs. TKR never=109^c^ | | TKR former=131^c^ vs. TKR never=233^c^ | |
| Total effect | 0.83 (0.62-1.07) | 100% | 1.45 (1.13-1.85) | 100% |
| Direct effect | 0.73 (0.57-0.94) | -^d^ | 1.38 (1.08-1.75) | 87% (60%-93%%) |
| Indirect effect via BMI | 1.14 (1.10-1.17) | -^d^ | 1.05 (1.03-1.07) | 13% (7%-40%) |

HR=hazard ratio, CI=confidence interval, BMI=body mass index.

^a^: Bootstrapping with 5000 iterations was used to calculate the uncertainty of the estimates.

^b^: On ln(HR) scale.

^c:^ The number of current, former or never smokers with TKR.

^d^: Percentages as proportion of the total effect are not given. Estimates were numerically unstable and therefore meaningless due to division by numbers (ln(HR_total effect_)) close to zero.
